# ZNF536 dysfunction enhances spontaneous differentiation of the SH-SY5Y cell line into a neuronal-like phenotype

**DOI:** 10.1192/j.eurpsy.2024.1224

**Published:** 2024-08-27

**Authors:** A. Kurishev, D. Abashkin, E. Marilovtseva, D. Karpov, V. Golimbet

**Affiliations:** Clinical Genetics Laboratoty, Mental Health Research Center, Moscow, Russian Federation

## Abstract

**Introduction:**

Schizophrenia (SZ) is a common psychiatric neurodevelopmental disorder with a complex genetic architecture. Genomic association studies indicate the involvement of transcription factors in the pathogenesis of SZ. A recent GWAS showed a significant association of ZNF536 with SZ. To date, the molecular functions of ZNF536 are poorly understood and its possible role in the pathogenesis of SZ is unclear.

**Objectives:**

The aim of this work was to develop a model cell line for study ZNF536-mediated pathogenic mechanisms associated with SZ.

**Methods:**

To assess the spatial interaction of ZNF536 with SZ risk loci, we used the Capture-C method. For ZNF536 deletion, SH-SY5Y was sequentially transduced with two lentiviral vectors. The first expressed Cas9 under the control of a tetracycline regulated promoter and the second expressed a pair of sgRNAs for ZNF536 deletion. Puromycin was used to select transduced cells. Stably transduced cells were then treated with oxytetracycline to induce Cas9 expression. In parallel, SH-SY5Y were transduced with lentiviral constructs of Cas9 and sgRNA carrying a spacer lacking targets in the human genome to obtain a negative control. Individual clones were obtained by the limiting dilution method. The ZNF536 deletion was confirmed by PCR and Sanger sequencing.

**Results:**

A spatial interaction of ZNF536 with SZ risk loci was found, suggesting its involvement in SZ pathogenesis. Using the CRISPR/Cas9 system, we obtained several clones with heterozygous deletion of ZNF536. We observed that their growth and proliferation were significantly slowed down. In addition, the mutant clones spontaneously differentiate into a neuron-like phenotype in low-serum medium.

**Conclusions:**

We established a cellular model to study ZNF536-mediated mechanisms associated with SZ.

**Disclosure of Interest:**

None Declared

